# A cross-sectional assessment of knowledge, attitudes and self-perceived effectiveness of complementary and alternative medicine among pharmacy and non-pharmacy university students

**DOI:** 10.1186/s12906-019-2503-y

**Published:** 2019-05-03

**Authors:** Mariam Ashraf, Hamid Saeed, Zikria Saleem, Hassaan Anwer Rathore, Fawad Rasool, Eman Tahir, Tooba Bhatti, Javeria Khalid, Iqra Bhatti, Ayesha Tariq

**Affiliations:** 1 Section of Clinical Pharmacy, Punjab University College of Pharmacy, University of the Punjab, Allama Iqbal Campus, Lahore, 54000 Pakistan; 2 Department of Pharmacy Practice, Rashid Latif College of Pharmacy, Lahore, 54000 Pakistan; 3 School of Pharmaceutical Sciences, Universiti Sains Malaysia, 11800 Penang, Malaysia; 4 Department of Pharmacy Practice, Faculty of Pharmacy, Bahauddin Zakariya University, Multan, Pakistan

**Keywords:** Complementary and alternative medicine, Pharmacy students, Non-pharmacy students, Knowledge, Attitude, Pakistan

## Abstract

**Background:**

Traditional medicine has always been Pakistan’s cultural heritage, providing health care to a large part of its population. Thus, we aimed to assess and compare the knowledge, attitude, and perception about complementary and alternative medicine (CAM) between pharmacy and non-pharmacy students, the results of which may be helpful in devising national health-education policy.

**Methods:**

A cross-sectional study was conducted by enrolling 937 students, pharmacy (437) and non-pharmacy (500), of Punjab University, Lahore. A self-administered questionnaire was used for data collection. Data were analyzed using SPSS. (IBM v22).

**Results:**

Data suggested that majority of students knew about the use of traditional herbs and considered massage (**P**: 84.4%, **NP**: 82%, *p* = 0.099), homeopathy, herbs (**P**: 86.5%, **NP**: 81%, *p* = 0.064], yoga [**P**: 357 (81.7%), **NP**: 84%), *p* = 0.42] and spiritual healing (**P**: 85.6%, **NP**: 86.2%, *p* = 0.55) as effective and least harmful CAM modalities. The pharmacy students had better knowledge about CAM modalities compared to non-pharmacy students. Despite utilizing non-reliable sources of CAM information and their belief that CAM is practiced by quacks, the majority of students had positive attitudes and perceptions about CAM usage. Students also believed that CAM had a positive impact on health outcomes [**P**: 3.19 ± 1.04, **NP**: 3.02 ± 1.09, *p* = 0.008] and acceded to include CAM in the pharmacy curriculum. However, non-pharmacy students scored higher in their beliefs that CAM usage should be discouraged due to the non-scientific basis of CAM (**P**: 3.04 ± 0.97, **NP**: 3.17 ± 1.02, *p* = 0.028) and a possible threat to public health (**P**: 3.81 ± 1.74, **NP**: 4.06 ± 1.56, *p* = 0.02). On the other hand, pharmacy students believed that patients might get benefits from CAM modalities (**P**: 4.31 ± 1.48, **NP**: 4.12 ± 1.45, *p* = 0.02). Majority of students perceived that spiritual healing is the most useful and safer CAM modality, while acupuncture (**P**: 25.4%, **NP**: 21.8%, *p* = 0.0005), hypnosis (**P**: 26.8%, **NP**: 19.6%, *p* = 0.001) and chiropractic (**P**: 18.8%, **NP**: 11.6%, *p* = 0.0005) were among the harmful ones.

**Conclusion:**

In conclusion, despite poor knowledge about CAM, students demonstrated positive attitudes and beliefs regarding CAM. They exhibited better awareness about yoga, spiritual healing/prayer, herbs, and massage. Students also showed willingness to advance their knowledge about CAM and favored its inclusion in the curriculum.

**Electronic supplementary material:**

The online version of this article (10.1186/s12906-019-2503-y) contains supplementary material, which is available to authorized users.

## Background

Since antiquity, different remedies have been used to correct and maintain overall health and wellbeing of humans. As the time advanced, some previously used traditional methods, falling outside the domain of allopathic medicine, have been modified into currently well-known health care approaches that are now collectively recognized as “complementary and alternative medicine” [1]. The global prevalence of complementary and alternative medicine (CAM) usage varies from 9.8 to 76% [2]. Yet in India only, more than 90% of the population depend on it for primary health care [3]. This growing acceptance of CAM has been attributed to its convenience, perceived efficacy, safety and affordability, affected by personal, religious, and spiritual beliefs [4–6]. In Africa, Asia and Latin America, around 80% of the population relies upon traditional medicine despite the availability of modern medicinal products [7]. Notable Asian countries include, India (90%) [3], Pakistan [8] and China (40%) [9].

In Pakistan, people have strong faith in complementary and alternative medicine [8], yet very little attention is paid to its formal education or inclusion in pharmacy and medical curricula. In this regard, Pakistani medicinal plants and herbs are specifically taught under pharmacognosy theme as part of the doctor of pharmacy (Pharm D) degree curriculum. Other alternative medicine systems such as Homeopathy, Ayurvedic and Uunani medicine are widely practiced in Pakistan but are taught as separate degree courses under Unani, Ayurvedic and Homeopathic act (UAH) 1965. [10, 11]. According to drug act 2012, pharmacists are permissible to deal with CAM. Similarly, the regulation of CAM is now under the jurisdiction of the drug regulatory authority of Pakistan (DRAP) according to DRAP act 2012. However, the combined efforts are lacking in order to utilize the CAM potential to its fullest to benefit the health care system. Nonetheless, the National Health Policy of 2001 has proposed amendments in Unani, Ayurvedic, and Homoeopathic (UAH) Act, 1965 to integrate degree and post-graduate level courses, which has now been implemented [10].

Numerous literature evidences suggest that herbal medicine, spiritual healing/prayer, massage-therapy, acupuncture, hypnosis, meditation, vitamins, and homeopathy are the most commonly known CAM modalities practiced in different regions of the world [12–14]. The use of CAM has increased remarkably in both developed and developing countries due to offhand affordability and accessibility along with better expectations regarding efficacy and safety profiles for treating various illness [4]. Several studies have reported high rates of CAM utilization among pharmacy, medical and nursing students [15–17]. Likewise, several literature reports also suggested higher prevalence of CAM product self-use (70%) among medical and pharmacy students [18–20]. It is also well documented that health management practices and choices, especially among pharmacy/medical students, are contrived during university times, which they continue in their professional lives ahead [21, 22]. On the other hand, it is equally important to explore CAM usage among non-medical students, since they are more vulnerable to inappropriate and incongruous use of CAM [23].

According to an estimate, the prevalence of CAM use is more than 70% in developing countries of Asian continent [24], while it was found to be 51.7% in Pakistan as stated in a population based survey conducted in 2009 [25]. The use of CAM in Pakistan is not only affected and actuated by culture, religion, access to health care providers and affordability but has also become a pivotal factor to be reckoned with our healthcare system due to widely trusted consumption of indigenous herbs as medicine [26]. Thus, despite continual increase and routine practice of CAM in Pakistan, CAM inclusion in medical and pharmacy curricula has not been evident probably because it is still considered inconsequential by allopathic health providers. This may be attributed to lack of scientific evidence-based promotion and utilization of CAM along with inabilities of allopathic health providers to respond to the queries related to safety, benefits and interactions of CAM products [15]. CAM usage is not specific to the pharmacy and medical students and many self-use practices and health-related recommending habits develop during university times. Therefore, it is pertinent to not only consider pharmacy students’ knowledge, attitudes and barriers towards the use of CAM but also evaluate non-pharmacy students who at times are more vulnerable to its inappropriate use. Thus, the present study was aimed at estimating and comparing the knowledge, beliefs, attitudes, self-perceived effectiveness and barriers of CAM among Pharmacy (P) & Non-Pharmacy (NP) university students.

## Methods

### Ethical approval

The Human Ethical Committee, University College of Pharmacy, University of the Punjab, Lahore, approved the study, reference number HEC/PUCP/099/2016. Verbal consent was obtained from all the participants.

### Study design

A descriptive, cross sectional study was conducted by sampling respondents from Pakistan’s oldest and biggest university, University of the Punjab, Lahore. The study was conducted from October 2016 to April 2017. Data was collected from both pharmacy and various non-pharmacy departments/disciplines (Fine arts, Language, Social and islamic studies, Social and administrative sciences, pure sciences and Engineering) of the university. Medical, dental and biological sciences students were excluded from the study for two main reasons; firstly, the objective of the study was to specifically compare the knowledge, attitudes, beliefs and self-perceived effectiveness between students acquiring pharmacy education with those acquiring Arts, Social studies, Languages, Islamic studies, Engineering, Business administration and computer education. Secondly, the commonality of the subjects (physiology, pharmacology and immunology) taught in the pharmacy, medical, dental and biological sciences disciplines would have caused redundancy in comparison of knowledge and perceptions of students from these educational backgrounds. Thus, it is likely that the students of these disciplines, if compared, might possess comparable information about CAM and are not among the group of students highly vulnerable to the irrational use of CAM. All the students were briefed about the nature and objectives of the research before administering the data collection tool. Data obtained from pharmacy and non-pharmacy students were segregated into separate arms for comparative analysis.

### Study population

A total of 937 students from the University of The Punjab (PU) were enrolled in the study. The sampling frame consisted of undergraduate and master students enrolled at the University. A sample size of 384 was estimated by Rao-soft software using 5% margin of error, 95% confidence interval with expected response of 50% for an expected University students’ population size of Lahore, i-e., approx. 2 million. However, to have a true representation of each group, more than 400 students were enrolled per group. A simple convenient sampling was used for the selection of study participants.

#### Inclusion criteria

All the students, enrolled in undergraduate or master degree program at the university either in pharmacy or non-pharmacy subjects (Fine arts, Language, Social and Islamic studies, Social and administrative sciences, pure sciences and Engineering), irrespective of age, gender, ethnicity, religion or social class. Only participants willing to take part in the study were enrolled.

#### Exclusion criteria

All undergraduate, post-graduate students of medical, dental and biological sciences departments and those not willing to participate in the study were excluded. Medical, dental and biological sciences students were excluded to clearly differentiate the opinions of students based on unrelated education, since medical, pharmaceutical, dental and biological sciences studies shared many common subjects as mentioned earlier.

### Data collection

Data collection was done by means of a comprehensive instrument of measure designed after extensive literature review [27–33] (Additional file 3 Annexure 1). The questionnaire was sent to subject experts/academicians for content validation, thereafter their expert opinion was incorporated to make the questionnaire more simple and objective driven. The reliability of the questionnaire was evaluated with Cronbach’s alpha (0.78) using SPSS version 22. Face validation of the questionnaire was done by conducting a pilot study on 20 students and the student’s feedback was incorporated in the final data collection form. The data obtained during the pilot study was not included in the final analysis. The self-administered questionnaire was distributed to selected students and were asked to submit the filled forms at the department admission office for final collection. A total of 1230 questionnaires were distributed out of which only 937 questionnaires were found to be complete. Data obtained from incomplete forms was not included in the final analysis. The questionnaire was outlined into the following **6** sections.

#### Section 1

Basic demographics, such as age, gender, marital status, the area of residence, living status, study area and others such as access to a family physician.

#### Section 2

Knowledge about 13 commonly known CAM modalities (acupuncture, aromatherapy, ayurveda, cupping, chiropractic, herbs, homeopathy, hypnosis, meditation, massage, oriental medicine, spiritual healing and yoga) was assessed using four options; never heard, heard but no knowledge, understand basic principles and pursued further knowledge (Additional file 3: Annexure 1). Knowledge related to use of CAM was estimated by additional 10 statements about certain CAM products such as mulethi (*Glycyrrhiza glabara*), joshanda, garlic (*Allium sativum*), ginseng (*Panax ginseng*) and *Ginkgo biloba* (Additional file: Annexure 1).

#### Section 3

Sources of CAM information, such as CAM professionals, health facilities, natural products professional journals, mass media, family & friends, internet or CAM web sites and training/apprenticeship with healers.

#### Section 4

Health related CAM beliefs were estimated utilizing a validated 10-items CAM health belief questionnaire (CHBQ) [34] based upon 7- point Likert scale from 1 = absolutely disagree to 7 = absolutely agree [34]. CHBQ were scored in such a way that a maximum score of 70 represents a strong positive attitude and 35 regarded as neutral, however minimum score of 10 is indicative of strong negative attitude. Additionally, 7 questions were added to estimate the use related CAM beliefs.

#### Section 5

This section consisted of twenty-eight statements to assess attitudes of respondents on a 5- point Likert scale (1 = strongly disagree to 5 = strongly agree) in addition to CHBQ items as it provided limited information about CAM health provision and practicing beliefs. Moreover, the additional items in section 5 were included to broaden the scope of documenting students’ opinions/attitudes towards CAM usage taking into account CAM education, CAM health provision and CAM rational practices.

#### Section 6

Self-perceived effectiveness of 13 CAM modalities.

### Data analysis

The data was analyzed using SPSS (IBM, version 22), unless otherwise stated. Descriptive analysis was performed to estimate the percentages and frequencies. Association of dependent variables including knowledge, attitude, beliefs and self-perceived effectiveness and independent variables, such as demographics were estimated using Pearson’s Chi-square. Non-parametric Manny-Whitney test was used to compare the difference between continuous variables. The normality of the data was determined by Shapiro-Wilk test, confirming that data did not represent a normal distribution pattern. An alpha value of 0.05 or less was considered statistically significant.

## Results

A total of 1230 questionnaires were distributed to the students, out of which 937 students responded with filled questionnaires making an overall response rate of 76.18%.

### Population basic demographics

Demographic profiles of respondents are summarized in Table 1. Out of 937 students, 437 (46.6%) belonged to the faculty of pharmacy and 500 (53.4%) from non-pharmacy departments (Table 1). Data suggested that the mean age in years of the respondents was 20.40 ± 1.85 years and out of total, 251 (26.8%) were male and 686 (73.2%) were female respondents. Only 2.9% of the students were married and 13.45% came from rural areas. The majority (76.5%) of students were day scholars and reported easy access to a family physician (70.8%) (Table 1).

**Table 1:** Population Basic Demographics

Characteristics	Pharmacy *n* = 437	Non-pharmacy *n* = 500	*p*-value
Age			
Mean ± SD	20.55 ± 1.95	20.27 ± 1.76	0.006*
Gender			
Male	88 (20.1%)	163 (32.6%)	0.0005**
Female	349 (79.9%)	337 (67.4%)	
Marital Status			
Single	428 (97.9%)	482 (96.4%)	0.160
Married	9 (2.1%)	18 (3.6%)	
Area Of Residence			
Urban	399 (91.3%)	412 (82.4%)	0.0005**
Rural	38 (8.7%)	88 (17.6%)	
Current Status			
Day scholar	314 (71.9%)	403 (80.6%)	0.002*
Boarder	123 (28.1%)	97 (19.4%)	
Access To Family Physician			
Easy	342 (78.3%)	321 (64.2%)	0.0005**
Difficult	95 (21.7%)	179 (35.8%)	

p-values: * 0.05–0.002, ** < 0.002

Noteworthy demographic variables with statistically significant differences between pharmacy and non-pharmacy students include, age (*p* = 0.006*), gender (*p* = 0.0005**), area of residence (*p* = 0.0005**) current status (*p* = 0.002*) and access to family physician (*p* = 0.0005**) (Table 1).

### Assessment of Student’s knowledge about CAM modalities

The pharmacy (**P**) students appeared to have greater knowledge/understanding about most of the CAM modalities compared to non-pharmacy (**NP**) students (Additional file 1: Table S1). Students of both the groups differed in their knowledge regarding CAM modalities (*p* = 0.0005), except for cupping (*p* = 0.165) and chiropractic (*p* = 0.422). More than 55% of the Pharmacy students had basic understanding about massage therapy (**P**: 62.9%, **NP**: 48.2%), herbs (**P:** 58.8%, **NP:** 40%), yoga (**P:** 55.8%, **NP:** 40.6%), spiritual healing (**P:** 58.6%, **NP:** 46.2%) and homeopathy (**P:** 51%, **NP:** 29.4%). On the other hand, more than 44% of non-pharmacy students never heard about chiropractic (**P:** 41.2%, **NP:** 44.6%), oriental medicine (**P:** 28.4%, **NP:** 55.8%) (Additional file 1: Table S1) and hypnosis (**P:** 19.9%, **NP:** 40%) (Additional file 1: Table S1). Moreover, more than 40% of the pharmacy students had just hearsay information about Acupuncture (46.9%) and Ayurveda (44.6%) but had little knowledge about these modalities. Majority of non-pharmacy students had no proper knowledge about homeopathy (47.8%), acupuncture (43.4%) and herbs (41.8%) although they had heard about these CAM modalities. Overall spiritual healing (**P:** 17.8%, **NP:** 22.6%) was pursued by greater number of students in both the groups (Additional file 1: Table S1).

### Student’s knowledge about specific CAM products

As shown in Table 2, most of the students had adequate knowledge about the use of joshanda in cold and flu (**P:** 92.2%, **NP:** 88.4%), use of Mulethi (*Glycyrrhiza glabara*) for sore throat and cough (**P:** 74.1%, **NP:** 67.2%) and use of garlic (*Allium sativum*) as lipid-lowering agent (**P:** 68.0%, **NP:** 51.8%). Moreover, students had no idea about the potential uses of ginseng, *Ginkgo biloba* and chiropractic, as well as about drug-herb interactions of mulethi (*Glycyrrhiza glabara*) (Table 2). Nearly 70% of the students imprecisely perceived herbal medicine to be safe with no side effects (Table 2). With respect to 10 knowledge statements in the questionnaire, only 4 were correctly marked by > 50% of pharmacy students, whereas only 3 statements were correctly marked by > 50% of non-pharmacy students (Table 2).

**Table 2:** Use Related CAM Knowledge

Statement (correct answer)	Use related CAM knowledge			*p*-value
	Correct	In-correct	Don’t know	
Herbal medicine is natural and therefore is safe, without side effects *(in-correct).*				
Pharmacy (437)	296 (67.7%)	122 (27.9%)	19 (4.3%)	0.0005**
Non-pharmacy (500)	380 (76.0%)	75 (15.0%)	45 (9.0%)	
Mulethi is commonly used herb for mild to moderate sore throat and cough *(correct).*				
Pharmacy (437)	324 (74.1%)	20 (4.6%)	93 (21.3%)	0.060
Non-pharmacy (500)	336 (67.2%)	33 (6.6%)	131 (26.2%)	
Mulethi may cause drug-herb interactions *(correct).*				
Pharmacy (437)	103 (23.6%)	74 (16.9%)	260 (59.5%)	0.117
Non-pharmacy (500)	116 (23.2%)	113 (22.6%)	271 (54.2%)	
Joshanda is commonly used for cold and flu symptoms *(correct).*				
Pharmacy (437)	403 (92.2%)	11 (2.5%)	23 (5.3%)	0.004*
Non-pharmacy (500)	422 (88.4%)	36 (7.2%)	22 (4.4%)	
Long-term use of Joshanda and mulethi may be harmful *(correct).*				
Pharmacy (437)	161 (36.9%)	103 (23.6%)	172 (39.4%)	0.007*
Non-pharmacy (500)	155 (31.0%)	164 (32.8%)	181 (36.2%)	
Garlic can lower blood lipid level *(correct).*				
Pharmacy (437)	297 (68.0%)	26 (5.9%)	114 (26.1%)	0.0005**
Non-pharmacy (500)	259 (51.8%)	43 (8.6%)	198 (39.6%)	
Ginseng can be used safely in people with high blood pressure *(in-correct).*				
Pharmacy (437)	93 (21.3%)	32 (7.3%)	312 (71.4%)	0.095
Non-pharmacy (500)	79 (15.8%)	41 (8.2%)	380 (76.0%)	
*Ginkgo biloba* is commonly used in people with Alzheimer’s disease *(correct).*				
Pharmacy (437)	88 (20.1%)	28 (6.4%)	321 (73.5%)	0.009*
Non-pharmacy (500)	65 (13.0%)	42 (8.4%)	393 (78.6%)	
Acupuncture can be used to decrease withdrawal symptoms and relieve pain *(correct).*				
Pharmacy (437)	276 (63.2%)	40 (9.2%)	121 (27.7%)	0.0005**
Non-pharmacy (500)	185 (37.0%)	47 (9.4%)	268 (53.6%)	
Chiropractic specializes in spinal manipulation and is used to treat low-back pain *(correct).*				
Pharmacy (437)	157 (35.9%)	34 (7.8%)	246 (56.3%)	0.003*
Non-pharmacy (500)	126 (25.2%)	41 (8.2%)	333 (66.6%)	

p-values: * 0.05–0.002, ** < 0.002

*CAM complementary and alternative medicine*

### Sources of CAM Information & Health Related CAM attitudes

The most commonly utilized sources of information among pharmacy and non-pharmacy students were family/friends and mass media (TV, newspapers, magazines, radio, wall chalking). Moreover, very few students gained CAM information by means of training/apprentice with healers (Additional file 2: Table S2).

Respondent’s attitude scored using a 5-point likert scale are summarized in Table 3. Mean attitude scores of less than 3 and more than 3 were considered negative and positive attitudes, respectively.

**Table 3:** Health Related Attitudes of Students towards CAM

Sr. #	Scores	Pharmacy *n* = 437	Non pharmacy *n* = 500	*p*-value
1	All practitioners of CAM should be medically qualified	3.87 ± 2.63	3.76 ± 1.17	0.896
2	I am interested in exploring new CAM modalities	3.29 ± 0.94	3.14 ± 1.00	0.038*
3	Women have more tendency to CAM than men	3.23 ± 0.98	3.22 ± 0.95	0.784
4	On an average, practitioners of CAM make less money than other doctors	3.45 ± 2.14	3.25 ± 1.01	0.084
5	I believe in alternative approaches in health area	3.40 ± 0.94	3.24 ± 0.99	0.006*
6	Most Practitioners of CAM receive a thorough training	3.16 ± 1.74	2.90 ± 1.09	0.008*
7	Treating a condition using CAM is safer than using modern methods	3.07 ± 1.01	3.01 ± 0.99	0.298
8	You need to be “gifted” to carry out CAM	3.04 ± 1.41	2.86 ± 1.08	0.026*
9	CAM has low status within medicine	3.31 ± 1.74	3.34 ± 0.95	0.135
10	CAM is only effective in treating minor complaints.	3.15 ± 1.00	3.24 ± 0.95	0.151
11	CAM is fairly unscientific.	3.04 ± 0.97	3.17 ± 1.02	0.028*
12	CAM has advanced considerably in recent years in understanding of illness and diseases	3.34 ± 1.74	3.15 ± 0.99	0.040*
13	Practitioners of CAM are more prepared to listen to their patients	3.19 ± 1.03	3.41 ± 0.94	0.002*
14	Patients on CAM hardly ever get better	2.89 ± 0.97	3.15 ± 0.99	0.0005**
15	Despite considerable research, there are few applicable results in CAM.	3.15 ± 0.94	3.30 ± 0.95	0.011*
16	CAM should be taught in medical school.	3.45 ± 1.74	3.46 ± 1.03	0.218
17	A surprising number of patients claim its effective at curing their illness	3.37 ± 0.96	3.19 ± 0.99	0.002*
18	CAM is more cost-effective than modern medicine	3.09 ± 1.43	3.12 ± 1.01	0.299
19	The reason for the success of CAM is mainly due to treating the whole person	3.26 ± 0.89	3.04 ± 1.09	0.001**
20	A doctor should know CAM methods	3.59 ± 1.65	3.52 ± 1.06	0.748
21	I believe that CAM may have positive effect on general health outcomes	3.19 ± 1.04	3.02 ± 1.09	0.008*
22	It is important to have a basic understanding of CAM before using them	3.60 ± 0.95	3.65 ± 0.99	0.209
23	Patients may disregard or even avoid doctors and health care professionals who do not understand their health beliefs.	3.39 ± 2.21	3.46 ± 1.11	0.020*
24	Herbal medicine is unsafe and ineffective.	2.54 ± 1.03	2.53 ± 1.06	0.821
25	Self-care and interest in our own health is one reason that people are drawn to CAM	3.26 ± 0.97	3.33 ± 0.96	0.393
26	Providing information about herbal medicine is part of a pharmacist’s professional responsibility.	3.43 ± 1.06	3.53 ± 0.93	0.206
27	Providing information about herbal medicine is part of a doctor’s professional responsibility.	3.27 ± 1.03	3.28 ± 1.07	0.900
28	It is important to consult a health professional before using CAM.	3.53 ± 0.98	3.62 ± 0.99	0.111

*Mann-Whitney U test were applied at a level of significance p ≤ 0.05*

*Responses were based on a 5 point Likert-type scale with 1 = Strongly Disagree 2 = Disagree 3 = Neutral 4 = Agree 5 = Strongly Agree*

*Mean Attitude scores greater than 3 (midpoint/neutral score) were treated as high and below 3 as low mean score*

Notable individual items exhibiting significant differences between pharmacy and non-pharmacy students included item # 2, exploring new CAM modalities (*p* = 0.038), item # 12, advancement in CAM (*p* = 0.04), item # 13, practitioners are more prepared and responsive (*p* = 0.002) and item# 21, positive impact of CAM on general health outcomes (*p* = 0.008) (Table 3).

Using the five-point likert scale, pharmacy students scored higher compared to non-pharmacy students for item # 5 (believe in alternative health approaches) (**P:** 3.40 ± 0.94, **NP:** 3.24 ± 0.99), and item # 4, (CAM practitioners make less money than doctors) (**P:** 3.45 ± 2.14, **NP:** 3.25 ± 1.01, *p* = 0.006) (Table 3). Conversely, non-pharmacy students scored higher for item # 13 (practitioners of CAM are more prepared to listen to their patients) (**P:** 3.19 ± 1.03, **NP:** 3.41 ± 0.94, *p* = 0.002) and item # 23 (patients may disregard or even avoid doctors and health care professionals who do not understand their health beliefs) (**P:** 3.39 ± 2.21, **NP:** 3.46 ± 1.11, *p* = 0.02) (Table 3).

### Use related CAM beliefs

In both groups of students, more than half (50%) believed that someone close to them had use CAM effectively for the treatment of an ailment (Table 4). This corroborated well with the prevalence of students in each group who believed in CAM efficacy (**P:** 80%, **NP:** 79.2%). Almost three-quarter of the students in both groups believed that CAM is practiced by quacks, with 34.1% pharmacy and 47.6% non-pharmacy students believed that only naive or gullible people use CAM (Table 4).

**Table 4:** Use Related CAM Beliefs Among Students

Questions	Use Related CAM- beliefs		*p*-values
	No	Yes	
Has somebody close to you had effective treatment from a complementary practitioner?			
Pharmacy (437)	182 (41.6%)	255 (58.3%)	0.027*
Non-pharmacy (500)	249 (49.8%)	251 (50.2%)	
Is it only naive/gullible people who go to complementary practitioners?			
Pharmacy (437)	288 (65.9%)	149 (34.1%)	0.0005**
Non-pharmacy (500)	262 (52.4%)	238 (47.6%)	
Do you believe there are many “quacks” in complementary medicine?			
Pharmacy (437)	116 (26.5%)	321 (73.5%)	0.299
Non-pharmacy (500)	118 (23.6%)	382 (76.4%)	
Do you believe the most important factor of any treatment is its efficacy for cure?			
Pharmacy (437)	75 (17.2%)	362 (82.8%)	0.153
Non-pharmacy (500)	104 (20.8%)	395 (79.2%)	
Do you believe complementary medicine is more holistic than orthodox medicine?			
Pharmacy (437)	219 (50.1%)	218 (49.9%)	0.0005*
Non-pharmacy (500)	320 (64.0%)	180 (36.0%)	
Does someone close to you use a complementary practitioner?			
Pharmacy (437)	202 (46.2%)	235 (53.8%)	0.396
Non-pharmacy (500)	245 (49.0%)	255 (51.0%)	
Do you believe that complementary medicine is better than medicine to treat psychological illness?			
Pharmacy (437)	245 (56.1%)	192 (43.9%)	0.048*
Non-pharmacy (500)	248 (49.6%)	252 (50.4%)	

p-values: * 0.05–0.002, ** < 0.002

*CAM complementary and alternative medicine*

### Students’ health related CAM beliefs

Using CAM health belief questionnaire (CHBQ), where mean attitude score exceeding mid-point = 3 represented a positive belief, mean attitude scores of pharmacy and non-pharmacy students were estimated and summarized in Table 5. Data demonstrated insignificant differences in overall CHBQ scores between pharmacy (44.54 ± 6.28) and non-pharmacy students (43.91 ± 6.73), (*p* = 0.087). However, significant differences between pharmacy and non-pharmacy students, when estimated for individual CHBQ items, such as “body is essentially self-healing and the task of a health care provider is to assist in the healing process” (**P:** 5.04 ± 1.57, **NP:** 4.89 ± 1.48, *p* = 0.01), “complementary therapies are a threat to public health” (**P:** 3.81 ± 1.74, **NP:** 4.06 ± 1.56, *p* = 0.02), “treatments not tested in scientifically recognized manners should be discouraged” (**P:** 2.94 ± 1.77, **NP:** 3.24 ± 1.81, *p* = 0.006), “effects of complementary therapies are usually like placebo effects” (**P:** 4.39 ± 1.65, **NP:** 4.05 ± 1.48, *p* = 0.001), “conventional medicines could benefit from ideas and methods from complementary medicine” (**P:** 4.31 ± 1.48, **NP:** 4.12 ± 1.45, *p* = 0.021) and that “most complementary therapies stimulate the body’s natural therapeutic powers” (**P:** 4.54 ± 1.54, **NP:** 4.39 ± 1.43, *p* = 0.032) (Table 5).

**Table 5:** CAM Health Belief Scores Using CHBQ Questionnaire

Sr.#	Statement	Pharmacy Mean ± SD	Non-pharmacy Mean ± SD	*p*-value
1	The physical and mental health are maintained by an underlying energy or vital force	4.49 ± 1.76	4.44 ± 1.69	0.549
2	Health and disease are a reflection of balance between positive life-enhancing forces and negative destructive forces.	5.01 ± 1.59	4.77 ± 1.52	0.002*
3	The body is essentially self-healing and the task of a health care provider is to assist in the healing process	5.04 ± 1.57	4.89 ± 1.48	0.010*
4	A patient’s symptoms should be regarded as a manifestation of general imbalance of dysfunction affecting the whole body	4.91 ± 1.51	4.68 ± 1.53	0.005*
5	A patient’s expectations, health beliefs and values should be integrated into the patient care process	5.08 ± 1.52	5.25 ± 1.66	0.010*
6	Complementary therapies are a threat to public health	3.81 ± 1.74	4.06 ± 1.56	0.020*
7	Treatments not tested in a scientifically recognized manner should be discouraged	2.94 ± 1.77	3.24 ± 1.81	0.006*
8	Effects of complementary therapies are usually the result of a placebo effect	4.39 ± 1.65	4.05 ± 1.48	0.001*
9	Complementary therapies include ideas and methods from which conventional medicine could benefit	4.31 ± 1.48	4.12 ± 1.45	0.021*
10	Most complementary therapies stimulate the body’s natural therapeutic powers	4.54 ± 1.54	4.39 ± 1.43	0.032*
	Total	44.54 ± 6.28	43.91 ± 6.73	0.087

*Responses of Item 6, 7 and 8 were reverse scored so a higher value indicated greater endorsement*

*An independent t test (Mann-Whitney test) were used at a level of significance p ≤ 0.05*

*Responses were based on a 7 point Likert-type scale with 1 = absolutely disagree to 7 = absolutely agree*

### Student’s perceived CAM effectiveness

Among all CAM modalities, spiritual healing (**P:** 85.6%, **NP:** 86.2%) was considered the most useful CAM modality by students of each group followed by herbs (**P:** 86.5%, **NP:** 81%), massage (**P:** 84.4%, **NP:** 82.0%), yoga (**P:** 81.7%, **NP:** 84%), aromatherapy (**P:** 81.2%, **NP:** 71.5%) and homeopathy (**P:** 76.7%, **NP:** 69.0%) (Table 6). On the other hand, acupuncture was considered the most harmful CAM modality by nearly one fourth of the pharmacy as well as non-pharmacy students (**P:** 25.4%, **NP:** 21.8%) followed by hypnosis (**P:** 26.8%, **NP:** 19.6%) and chiropractic (**P:** 18.8%, **NP:** 11.6%). Furthermore, aromatherapy (**P:** 3.9%, **NP:** 4.8%), spiritual healing (**P:** 4.6%, **NP:** 5.6%), herbs (**P:** 4.6%, **NP:** 7.4%) and yoga (**P:** 3.9%, **NP:** 4.4%) were considered as the least harmful CAM modalities. Interestingly, more than 40% of non-pharmacy students had no opinion about acupuncture, chiropractic and oriental medicine (Table 6).

**Table 6:** Student’s Perceived CAM Effectiveness

CAM Modality	Perceived CAM Effectiveness			*p*-value
	Harmful	Useful	No opinion	
Acupuncture				
Pharmacy (437)	111 (25.4%)	225 (51.5%)	101 (23.1%)	0.0005*
Non-pharmacy (500)	109 (21.8%)	170 (34.0%)	221 (44.2%)	
Aromatherapy				
Pharmacy (437)	17 (3.9%)	355 (81.2%)	65 (14.9%)	0.0005*
Non-pharmacy (500)	24 (4.8%)	670 (71.5%)	226 (24.1%)	
Ayurveda				
Pharmacy (437)	35 (8.0%)	293 (67.0%)	109 (24.9%)	0.002*
Non-pharmacy (500)	33 (6.6%)	289 (57.8%)	178 (35.6%)	
Cupping				
Pharmacy (437)	64 (14.6%)	256 (58.6%)	117 (26.8%)	0.056*
Non-pharmacy (500)	57 (11.4%)	276 (55.2%)	167 (33.4%)	
Chiropractic				
Pharmacy (437)	82 (18.8%)	216 (49.4%)	139 (31.8%)	0.0005*
Non-pharmacy (500)	58 (11.6%)	197 (39.4%)	245 (49.0%)	
Herbs				
Pharmacy (437)	20 (4.6%)	378 (86.5%)	39 (8.9%)	0.064
Non-pharmacy (500)	37 (7.4%)	405 (81.0%)	58 (11.6%)	
Homeopathy				
Pharmacy (437)	44 (10.1%)	335 (76.7%)	58 (13.3%)	0.010*
Non-pharmacy (500)	82 (16.4%)	345 (69.0%)	73 (14.6%)	
Hypnosis				
Pharmacy (437)	117 (26.8%)	164 (37.5%)	156 (35.7%)	0.001*
Non-pharmacy (500)	98 (19.6%)	160 (32.0%)	242 (48.4%)	
Meditation				
Pharmacy (437)	54 (12.4%)	286 (65.4%)	97 (22.2%)	0.0005*
Non-pharmacy (500)	40 (8.0%)	282 (56.4%)	178 (35.6%)	
Massage				
Pharmacy (437)	28 (6.4%)	369 (84.4%)	40 (9.2%)	0.099
Non-pharmacy (500)	24 (4.8%)	410 (82.0%)	66 (13.2%)	
Oriental medicine				
Pharmacy (437)	36 (8.2%)	221 (50.6%)	180 (41.2%)	0.0005*
Non-pharmacy (500)	31 (6.2%)	176 (35.2%)	293 (58.6%)	
Spiritual healing				
Pharmacy (437)	20 (4.6%)	374 (85.6%)	43 (9.8%)	0.553
Non-pharmacy (500)	28 (5.6%)	431 (86.2%)	41 (8.2%)	
Yoga				
Pharmacy (437)	17 (3.9%)	357 (81.7%)	63 (14.4%)	0.419
Non-pharmacy (500)	22 (4.4%)	420 (84.0%)	58 (11.6%)	

p-values: * 0.05–0.002, ** < 0.002

*CAM complementary and alternative medicine*

## Discussion

Lately, complementary and alternative medicine (CAM) has reverted back from ancient civilizations to the modern era. As of today, more than 80% of the developing world’s population still depends upon CAM, while half in the developed world use CAM [24]. Nevertheless, the nature and use of CAM may vary in different regions depending upon local culture and environment. Thus, Pakistan being an Asian country has its own perspectives with respect to CAM use [11]. The data from the present study demonstrated that pharmacy students had better knowledge with more positive beliefs and attitudes towards CAM compared to non-pharmacy students. The students also had sound knowledge about locally used CAM treatments including mulethi (*Glycyrrhiza glabara*), joshanda and garlic (*Allium sativum*).

Our study highlighted that of all the CAM modalities, massage, spiritual healing, yoga, herbs and homeopathy were the most commonly known CAM modalities among students in both the groups. This finding is similar to a previous report by Gelaw et al and his co-workers demonstrating that herbal medicine, massage and spiritual healing are the most commonly known CAM practices among students [7]. Other studies supporting this view have been reported on students from Kuwait [5], Syria [4], Ghana [20] and Czech Republic [29]. Likewise, studies from Malaysia, Syria, Turkey have also supported our findings that massage, spiritual healing/prayers and homeopathy were commonly used CAM modalities among pharmacy and non-pharmacy students [12, 27, 29, 35]. These data suggested that CAM modalities, yoga, massage, herbs and spiritual healing are well acquainted CAM modalities among students of Arab, European, African and Asian origin, and are frequently practiced as well. Moreover, frequent interactions of pharmacy students with medicinal plants during their studies and frequent utilization of herbs for several ailments by family members and friends in a Pakistani society might be the reason for better knowledge and positive attitude among students towards herbs.

Conversely, oriental medicine and chiropractic remained the least known modalities for both pharmacy and non-pharmacy students – a trend that is consistent with the findings of a study from Singapore on medical students [36]. Data further suggested that the students of Pakistan were unfamiliar with oriental medicine and chiropractic therapy. This could be due to several reasons, i-e., Chinese and Western origins of these modalities, these modalities were rarely or never discussed among family members, friends and health providers, there is no formal education for Pharm D (doctor of pharmacy) students regarding CAM and almost non-existent or a very few practicing chiropractic and oriental medicine professionals in Pakistan. Similarly, massage was considered a useful common practice by the students because it is part of the local tradition. In this context, studies on Bangladeshi, Saudi and Australian students demonstrated better knowledge about massage and the majority of students were in favor of its use [21]. Likewise, homeopathy is also a well-known system in Pakistan owing to the large number of Homeopathic clinics and shops [11]. The improved knowledge and usage of spiritual healing/prayer among pharmacy and non-pharmacy students might be attributed to its routine usage in the community, although the keen interest/belief in its efficacy relates to the religious affiliations of the Pakistani community. On the other hand, higher knowledge about yoga among students, not routinely practiced in the Pakistani community, a tradition of the neighboring country, i-e. India, might be due to the influence of Indian culture transmitted via social and entertainment media. Despite easy access, overall students, particularly non-pharmacy, exhibited minimal interest in pursuing CAM as reported previously [31]. This is probably due to heavy study load on pharmacy students or minimal interaction of non-pharmacy students with sciences entailing CAM education.

Poor or insufficient knowledge about herbs has been reported among pharmacy students of Abbottabad and Hazara region of Pakistan [37]. In the present study, we also observed that more than half of the students from Lahore, both pharmacy and non-pharmacy, exhibited adequate knowledge about joshanda, mulethi (*Glycyrrhiza glabara*) and garlic (*Allium sativum*) - the highly popular and easily available herbs with frequent consumption in Lahore. Moreover, despite no formal education about CAM and unsatisfactory knowledge, pharmacy students demonstrated better practical knowledge about herbs compared to non-pharmacy students. This might be due to greater educational exposure of pharmacists with drugs and related information compared to non-pharmacy students.

Influence of family and culture, particularly in Asian population, regarding the use of CAM has been reported previously [38]. In complete agreement to this fact, our data suggested that both pharmacy and non-pharmacy students considered family, friends and mass media as the most common sources of CAM information. This is in line with previous findings from Pakistan [31], Thailand [39], Saudi Arabia [40] and Turkey **[12].** This data suggested that despite greater chances of misleading information and lack of trustworthy information, the above-mentioned sources were frequently utilized by pharmacy students.

Several lines of literature evidences suggested that students from United States, Saudi Arabia and Malaysia exhibited positive attitudes and beliefs towards CAM usage [19, 40, 41]. In this context, our findings also suggested that pharmacy students considered CAM more holistic than conventional medicine and believed in the advancements of CAM with earnest desire to explore more about it. Nevertheless, the students also believed that CAM should be made more scientific. Many literature reports suggested that pharmacy students supported the inclusion of CAM in their curriculum to better guide and practice CAM in rendering professional health services [4, 15, 33]. The findings from the present study and those reported previously suggested that students gained much of their information about CAM from non-scientific sources. Nonetheless, both the students, pharmacy and non-pharmacy, favored the inclusion of CAM in formal degree programs, such as pharmacy and medicine. This highlights the need to revise the academic curricula and health policies to regulate and standardize health care practices of CAM to ensure public protection.

Both pharmacy and non-pharmacy students perceived that aromatherapy, herbs, massage, spiritual healing and yoga are useful CAM modalities with better efficacy [5, 17, 42]. High acceptance and positive perception about the effectiveness of these modalities may be linked to religious and cultural norms being practiced by the elders who inculcated the beliefs of safety and efficacy of these modalities in their decedents by communicating personal experiences and exposing their children to practicing professionals.

Additionally, the students, pharmacy and non-pharmacy, strongly believed that patient’s expectations, health beliefs and values must be incorporated into the patient care process. Since improper attention by medical doctors to the patients and tenuous pharmacist-patient interactions might pave the way for many patients to consult CAM providers. In this regard an undeniable fact is the diversity of health-seeking behaviors in Pakistan, having a pluralistic health care system, where people have been consulting CAM providers for ages and will continue to seek CAM treatments for various reasons [26]. Therefore, it is imperative to bring CAM healers into main stream of the health care system by implementing integrative medicine approaches in education and health sector. This can be achieved by combining CAM and conventional medicine approaches in providing health care services in a clinical setting. In this context, attempts were made to bring CAM into mainstream national education and health policy. Thus, drug regulatory authority of Pakistan promulgated DRAP act 2012 with the aim to register all the CAM practitioners and to regulate nutraceuticals, followed by initiative of Pakistan’s national health policy to include Tibb (herbal medicine) in post-graduate education. Yet, many students believed that alternative approaches are being practiced by quacks without any research assertions with almost no proof even from the old testaments. Additionally, the CAM providers lack proper stock maintenance and storage conditions that may threaten the efficacy of their products if stocked for a longer period of time. Therefore, the government should implement the CAM regulatory policies under DRAP act 2012 to ensure proper provision and distribution of CAM in preventing un-authorized access and handling of CAM. The government should also empower CAM providers through training and CAM-friendly transition policies by helping them to develop proper stock maintenance and storage conditions. Additionally, an integrative medicine approach must be initiated, intermingling conventional medicine and CAM, which can only be guaranteed if physicians and CAM healers work in collaboration to ensure that all practices of CAM comply with professional, ethical and practice standards. Thus, among others, the sole major challenge is to initiate a policy document that ensures collaborative working relationship among allopathic conventional physician, pharmacist and CAM providers, since at the moment the relationship between them is of rivalry and animosity. Thus, to start with, a primary care model can be devised where conventional (allopathic) medical care and CAM care can be housed in the same setting.

### Limitations

Our study has several limitations; a cross-sectional study design, thus factors affecting student’s responses cannot be studied over time. Additionally, the knowledge as well as attitudes of the students regarding CAM may change over time, therefore, a longitudinal study should be performed to better explain these behaviors. Results of this survey only represent the opinions of the students belonging to University of The Punjab, Lahore, Pakistan which cannot be generalized to other universities of Lahore or Pakistan. The findings on CAM are based on student’s views whereas the vision of CAM practitioners has not been described who might have different opinions.

## Conclusion

Data suggested that both pharmacy and non-pharmacy students had favorable attitudes and beliefs towards CAM with better understanding and self-perceived effectiveness about the most commonly known CAM modalities, such as massage, homeopathy, spiritual healing/prayer, yoga and herbs. While oriental medicine and chiropractic therapy are the most unfamiliar CAM modalities among the students. Surprisingly, students exhibited improved knowledge about, yoga, not frequently practiced in Pakistan, probably due to cross border cultural influence, i-e., from India. Despite poor practical knowledge about CAM, mostly build upon unreliable sources of information, many students agreed to include CAM in pharmacy/medical curriculum to advance their knowledge about CAM and to confer more scientific logic behind its use. Thus, it is imperative to include CAM advance and basic education in pharmacy/medical and non-medical sciences disciplines, respectively. Moreover, public educational and awareness programs should be initiated at various health care settings, primarily those utilizing CAM modalities, such as CAM dawa khana’s (CAM dispensaries), community medicine outlets, and primary health centers.

## Additional files

Additional file 1: **Table S1.** Student’s Knowledge about CAM Modalities (DOCX 17 kb)

Additional file 2: **Table S2.** Sources of CAM information (DOCX 20 kb)

Additional file 3: **Annexure 1.** Study Questionnaire (DOCX 31 kb)

## Abbreviations

CAM: Complementary and alternative medicine
CHBQ: CAM health belief questionnaire
DRAP: Drug regulatory authority of Pakistan
UAH: Unani, Ayurvedic and Homeopathic act
