# Supplementary material for: A cross-sectional assessment of knowledge, attitudes and self-perceived effectiveness of complementary and alternative medicine among pharmacy and non-pharmacy university students
Source: BMC Complement Altern Med. 2019 May 3;19:95. doi: 10.1186/s12906-019-2503-y (PMC6500055; doi:10.1186/s12906-019-2503-y)
Supplement: Supplementary file 1 — Table S1. Student’s Knowledge about CAM Modalities (DOCX 17 kb) [file 12906_2019_2503_MOESM1_ESM.docx]

Table S1. Student’s Knowledge about CAM Modalities

| **CAM modality** | **Never heard** | **Heard but no knowledge** | **Understand basic principles** | **Pursued further knowledge** | ***p*-value** |
| --- | --- | --- | --- | --- | --- |
| **Acupuncture**  Pharmacy (437)  Non-pharmacy (500) | 40 (9.2%)  189 (37.8%) | 205 (46.9%)  217 (43.4%) | 179 (41.0%)  81 (16.2%) | 13 (3.0%)  13 (2.6%) | 0.0005** |
| **Aromatherapy**  Pharmacy (437)  Non-pharmacy (500) | 83 (19.0%)  186 (37.2%) | 166 (38.0%)  183 (36.6%) | 174 (39.8%)  114 (22.8%) | 14 (3.2%)  17 (3.4%) | 0.0005** |
| **Ayurveda**  Pharmacy (437)  Non-pharmacy (500) | 76 (17.4%)  155 (31.0%) | 195 (44.6%)  178 (35.6%) | 147 (33.6%)  130 (26.0%) | 19 (4.3%)  37 (7.4%) | 0.0005** |
| **Cupping**  Pharmacy (437)  Non-pharmacy (500) | 118 (27.0%)  143 (28.6%) | 131 (30.0%)  175 (35.0%) | 159 (36.4%)  149 (29.8%) | 29 (6.6%)  33 (6.6%) | 0.165 |
| **Chiropractic**  Pharmacy (437)  Non-pharmacy (500) | 180 (41.2%)  223 (44.6%) | 154 (35.2%)  152 (30.4%) | 81 (18.5%)  102 (20.4%) | 22 (5.0%)  23 (4.6%) | 0.422 |
| **Herbs**  Pharmacy (437)  Non-pharmacy (500) | 8 (1.8%)  32 (6.4%) | 128 (29.3%)  209 (41.8%) | 257 (58.8%)  200 (40.0%) | 44 (10.1%)  59 (11.8%) | 0.0005** |
| **Homeopathy**  Pharmacy (437)  Non-pharmacy (500) | 17 (3.9%)  62 (12.4%) | 158 (36.2%)  239 (47.8%) | 223 (51.0%)  147 (29.4%) | 39 (8.9%)  52 (10.4%) | 0.0005** |
| **Hypnosis**  Pharmacy (437)  Non-pharmacy (500) | 87 (19.9%)  200 (40.0%) | 149 (34.1%)  195 (9.0%) | 171 (39.1%)  89 (17.8%) | 30 (6.9%)  16 (3.2%) | 0.0005** |
| **Meditation**  Pharmacy (437)  Non-pharmacy (500) | 68 (15.6%)  161 (32.2%) | 166 (38.0%)  152 (30.4%) | 170 (38.9%)  149 (29.8%) | 33 (7.6%)  38 (7.6%) | 0.0005** |
| **Massage**  Pharmacy (437)  Non-pharmacy (500) | 9 (2.1%)  16 (3.2%) | 87 (19.9%)  157 (31.4%) | 275 (62.9%)  241 (48.2%) | 66 (15.1%)  86 (17.2%) | 0.0005** |
| **Oriental medicine**  Pharmacy (437)  Non-pharmacy (500) | 124 (28.4%)  279 (55.8%) | 163 (37.3%)  117 (23.4%) | 129 (29.5%)  77 (15.4%) | 21 (4.8%)  27 (5.4%) | 0.0005** |
| **Spiritual healing**  Pharmacy (437)  Non-pharmacy (500) | 21 (4.8%)  15 (3.0%) | 82 (18.8%)  141 (28.2%) | 256 (58.6%)  231 (46.2%) | 78 (17.8%)  113 (22.6%) | 0.0005** |
| **Yoga**  Pharmacy (437)  Non-pharmacy (500) | 12 (2.7%)  29 (5.8%) | 125 (28.6%)  185 (37.0%) | 244 (55.8%)  203 (40.6%) | 56 (12.8%)  83 (16.6%) | 0.0005** |
| ***p-values:*** ** 0.05 – 0.002, ** < 0.002*  ***CAM*** *= complementary and alternative medicine* | | | | | |
