# Supplementary material for: A cross-sectional assessment of knowledge, attitudes and self-perceived effectiveness of complementary and alternative medicine among pharmacy and non-pharmacy university students
Source: BMC Complement Altern Med. 2019 May 3;19:95. doi: 10.1186/s12906-019-2503-y (PMC6500055; doi:10.1186/s12906-019-2503-y)
Supplement: Supplementary file 2 — Table S2. Sources of CAM information (DOCX 20 kb) [file 12906_2019_2503_MOESM2_ESM.docx]

Table S2. Sources of CAM information

| **CAM Modalities** | **CAM professionals** | **Health facilities** | **Professional journals** | **Mass media** | **Family & friends** | **Internet or CAM web sites** | **Training/**  **Apprentice** | **None** | ***p*- values** |
| --- | --- | --- | --- | --- | --- | --- | --- | --- | --- |
| **Acupuncture**  Pharmacy (437)  Non-pharmacy (500) | 30 (6.9%)  34 (6.8%) | 94 (21.5%)  62 (12.4%) | 15 (3.4%)  22 (4.4%) | 133 (30.4%)  120 (24.0%) | 72 (16.5%)  73 (14.6%) | 49 (11.2%)  39 (7.8%) | 6 (1.4%)  6 (1.2%) | 38 (8.7%)  144 (28.8%) | 0.0005** |
| **Aromatherapy**  Pharmacy (437)  Non-pharmacy (500) | 21 (4.8%)  20 (4.0%) | 59 (13.5%)  37 (7.4%) | 51 (11.7%)  49 (9.8%) | 84 (19.2%)  107 (21.4%) | 82 (18.8%)  98 (19.6%) | 55 (12.6%)  36 (7.2%) | 4 (0.9%)  1 (0.2%) | 81 (18.5%)  152 (30.4%) | 0.0005** |
| **Ayurveda**  Pharmacy (437)  Non-pharmacy (500) | 23 (5.3%)  20 (4.0%) | 38 (8.7%)  26 (5.2%) | 50 (11.4%)  61 (12.2%) | 78 (17.8%)  90 (18%) | 145 (33.2%)  142 (28.4%) | 22 (5.0%)  31 (6.2%) | 4 (0.9%)  2 (0.4%) | 77 (17.6%)  128 (25.6%) | 0.034* |
| **Cupping**  Pharmacy (437)  Non-pharmacy (500) | 19 (4.3%)  17 (3.4%) | 39 (8.9%)  40 (8.0%) | 11 (2.5%)  39 (7.8%) | 91 (20.8%)  108 (21.6%) | 111 (25.4%)  127 (25.4%) | 44 (10.1%)  53 (10.6%) | 5 (1.1%)  11 (2.2%) | 117 (26.7%)  105 (21.0%) | 0.016* |
| **Chiropractic**  Pharmacy (437)  Non-pharmacy (500) | 24 (5.5%)  19 (3.8%) | 52 (11.9%)  40 (8.0%) | 24 (5.5%)  30 (6.0%) | 48 (11.0%)  94 (18.8%) | 72 (16.5%)  94 (18.8%) | 29 (6.6%)  47 (9.4%) | 8 (1.8%)  8 (1.6%) | 180 (41.2%)  168 (33.6%) | 0.004* |
| **Herbs**  Pharmacy (437)  Non-pharmacy (500) | 26 (5.9%)  19 (3.8%) | 76 (17.4%)  52 (10.4%) | 91 (20.8%)  69 (13.8%) | 60 (13.7%)  141 (28.2%) | 136 (31.1%)  159 (31.8%) | 36 (8.2%)  33 (6.6%) | 6 (1.4%)  7 (1.4%) | 6 (1.4%)  20 (4.0%) | 0.0005** |
| **Homeopathy**  Pharmacy (437)  Non-pharmacy (500) | 22 (5.0%)  23 (4.6%) | 110 (25.2%)  76 (15.2%) | 60 (13.7%)  33 (6.6%) | 53 (12.1%)  126 (25.2%) | 139 (31.8%)  171 (34.2%) | 28 (6.4%)  35 (7.0%) | 7 (1.6%)  5 (1.0%) | 18 (4.1%)  31 (6.2%) | 0.0005** |
| **Hypnosis**  Pharmacy (437)  Non-pharmacy (500) | 26 (5.9%)  22 (4.4%) | 66 (15.1%)  48 (9.6%) | 19 (4.3%)  16 (3.2%) | 114 (26.1%)  141 (28.2%) | 59 (13.5%)  63 (12.6%) | 56 (12.8%)  67 (13.4%) | 4 (0.9%)  4 (0.8%) | 93 (21.3%)  139 (27.8%) | 0.090 |
| **Meditation**  Pharmacy (437)  Non-pharmacy (500) | 17 (3.9%)  11 (2.2%) | 31 (7.1%)  23 (4.6%) | 21 (4.8%)  35 (7.0%) | 121 (27.7%)  118 (23.6%) | 123 (28.1%)  111 (22.2%) | 46 (10.5%)  60 (12.0%) | 12 (2.7%)  11 (2.2%) | 66 (15.1%)  131 (26.2%) | 0.001** |
| **Massage**  Pharmacy (437)  Non-pharmacy (500) | 38 (8.7%)  29 (5.8%) | 66 (15.1%)  53 (10.6%) | 29 (6.6%)  28 (5.6%) | 99 (22.7%)  171 (34.2%) | 157 (35.9%)  166 (33.2%) | 25 (5.7%)  38 (7.6%) | 12 (2.7%)  10 (2.0%) | 11 (2.5%)  5 (1.0%) | 0.002* |
| **Oriental medicine**  Pharmacy (437)  Non-pharmacy (500) | 29 (6.6%)  25 (5.0%) | 52 (11.9%)  30 (6.0%) | 44 (10.1%)  25 (5.0%) | 79 (18.1%)  118 (23.6%) | 76 (17.4%)  48 (9.6%) | 31 (7.1%)  30 (6.0%) | 2 (0.5%)  5 (1.0%) | 124 (28.4%)  219 (43.8%) | 0.0005** |
| **Spiritual healing**  Pharmacy (437)  Non-pharmacy (500) | 24 (5.5%)  19 (3.8%) | 32 (7.3%)  22 (4.4%) | 26 (5.9%)  32 (6.4%) | 75 (17.2%)  86 (17.2%) | 228 (52.2%)  304 (60.8%) | 17 (3.9%)  21 (4.2%) | 16 (3.7%)  11 (2.2%) | 19 (4.3%)  5 (1.0%) | 0.006* |
| **Yoga**  Pharmacy (437)  Non-pharmacy (500) | 22 (5.0%)  19 (3.8%) | 48 (11.0%)  26 (5.2%) | 27 (6.2%)  35 (7.0%) | 153 (35.0%)  203 (40.6%) | 102 (23.3%)  113 (22.6%) | 58 (13.3%)  75 (15.0%) | 15 (3.4%)  13 (2.6%) | 12 (2.7%)  16 (3.2%) | 0.050* |
| ***p-values:*** ** 0.05 – 0.002, ** < 0.002*  ***CAM*** *= complementary and alternative medicine* | | | | | | | | | |
