# Supplementary material for: A cross-sectional assessment of knowledge, attitudes and self-perceived effectiveness of complementary and alternative medicine among pharmacy and non-pharmacy university students
Source: BMC Complement Altern Med. 2019 May 3;19:95. doi: 10.1186/s12906-019-2503-y (PMC6500055; doi:10.1186/s12906-019-2503-y)
Supplement: Supplementary file 3 — Annexure 1. Study Questionnaire (DOCX 31 kb) [file 12906_2019_2503_MOESM3_ESM.docx]

**Annexure 1**

**Study Questionnaire**

(*A Cross-sectional Assessment of Knowledge, Attitudes and Self-Perceived Effectiveness of CAM among Pharmacy and Non-Pharmacy University Students*)

**SECTION: 1 DEMOGRAPHIC CHARACTERISTICS**

**Age**: _____ **Gender**: ☺ Male ☺ Female **Marital Status**: ☺ Single ☺ Married

**Area of Residence**: ☺ Urban ☺ Rural **Current Status**: ☺ Day Scholar ☺ Hostel lite

**Study Area**: ___________ **Professional Year**: __________

**Access to Family Physician**: ☺ Easy ☺ Difficult

**SECTION: 2 KNOWLEDGE ASSESMENT (**CAM modalities**)**

1. Have you ever heard about any of the following CAM modalities? (select your option by tick mark)

| **CAM modality** | **Never heard** | **Heard but no knowledge** | **Understand basic principles** | **Pursued further knowledge** |
| --- | --- | --- | --- | --- |
| **Acupuncture**  (*inserting fine needles through the skin at specific points to relieve pain or promote healing)* |  |  |  |  |
| **Aromatherapy**  (*use of essential oils of plants for medicinal purposes*) |  |  |  |  |
| **Ayurveda**  *(hikmat)* |  |  |  |  |
| **Cupping/Hijama**  (*the process of drawing blood from the body by placing special cups on skin)* |  |  |  |  |
| **Chiropractic**  *(a method of treating people who are sick or in pain by pushing and moving bones in the spine and joints)* |  |  |  |  |
| **Herbs**  *(Herbal medicine)* |  |  |  |  |
| **Homeopathy**  *(Used to stimulate the body's own healing process)* |  |  |  |  |
| **Hypnosis**  *(an artificially induced trance state resembling sleep)* |  |  |  |  |
| **Meditation / Muraqba**  *(act of spending time in quiet thought either as a*[*religious*](http://dictionary.cambridge.org/dictionary/english/religious) [*activity*](http://dictionary.cambridge.org/dictionary/english/activity)*or relaxation)* |  |  |  |  |
| **Massage**  (*Maalish)* |  |  |  |  |
| **Oriental medicine**  *(The treatment which incorporates the use of acupuncture, herbs, heat therapy, physical therapies, and lifestyle changes to restore balance of Qi or Chee)* |  |  |  |  |
| **Spiritual healing**  *(prayer)* |  |  |  |  |
| **Yoga**  *(physical, mental, and spiritual practices to experience inner peace)* |  |  |  |  |

1. **Use related CAM knowledge** (select your option by tick mark)

| **Statement** | **Correct** | **In-correct** | **Don’t know** |
| --- | --- | --- | --- |
| Herbal medicine is natural and therefore is safe, without side effects |  |  |  |
| Mulethi is commonly used herb for mild to moderate sore throat and cough |  |  |  |
| Mulethi may cause drug-herb interactions |  |  |  |
| Joshanda is commonly used for cold and flu symptoms |  |  |  |
| Long-term use of Joshanda and mulethi is not recommended |  |  |  |
| Garlic can lower blood lipid level |  |  |  |
| Ginseng can be used safely in people with high blood pressure |  |  |  |
| Ginkgo biloba is commonly used in people with Alzheimer’s disease |  |  |  |
| Acupunture can be used to decrease withdrawal symptoms and relieve pain |  |  |  |
| Chiropractic specializes in spinal manipulation and is used to treat low-back pain |  |  |  |

**SECTION: 3** **SOURCES OF CAM INFORMATION**

From which source, you got knowledge about following CAM modalities?

| **CAM Modalities** | CAM professionals | Health Facilities (hospital,  Pharmacy, diagnostic lab, Clinic) | Natural Product professional journals | Mass media (TV, newspapers, magazines, radio, wall chalking) | Family & Friends | Internet  or CAM Web sites | Training/  Apprentice with healers | None |
| --- | --- | --- | --- | --- | --- | --- | --- | --- |
| **Acupuncture** (*Inserting fine needles through the skin)* |  |  |  |  |  |  |  |  |
| **Aromatherapy (***use of essential oils of plants)* |  |  |  |  |  |  |  |  |
| **Ayurveda**  **(***hikmat)* |  |  |  |  |  |  |  |  |
| **Cupping/Hijama**  *(Placing cups on skin to draw blood)* |  |  |  |  |  |  |  |  |
| **Chiropractic** *(Treating by pushing and moving bones in the spine and joints)* |  |  |  |  |  |  |  |  |
| **Herbs**  *( herbal medicine)* |  |  |  |  |  |  |  |  |
| **Homeopathy**  *(Stimulating body's own healing process)* |  |  |  |  |  |  |  |  |
| **Hypnosis**  *(An artificially induced trance state resembling sleep)* |  |  |  |  |  |  |  |  |
| **Meditation/ Muraqba**  *(Act of spending time in quiet thought)* |  |  |  |  |  |  |  |  |
| **Massage**  (*Maalish)* |  |  |  |  |  |  |  |  |
| **Oriental medicine**  *(Aims to restore balance of life energy)* |  |  |  |  |  |  |  |  |

| **Spiritual healing** *(Prayer)* |  |  |  |  |  |  |  |  |
| --- | --- | --- | --- | --- | --- | --- | --- | --- |
| **Yoga** *(practices to experience inner peace)* |  |  |  |  |  |  |  |  |

**SECTION: 4** **Health Related CAM Beliefs** (Select your option by tick mark)

1. CAM Health Belief Questionnaire (CHBQ)

| **Statement** | **Absolutely disagree** | **Disagree** | **Somewhat disagree** | **Neutral** | **Somewhat agree** | **Agree** | **Absolutely agree** |
| --- | --- | --- | --- | --- | --- | --- | --- |
| The physical and mental health are maintained by an underlying energy or vital force |  |  |  |  |  |  |  |
| Health and disease are a reflection of balance between positive life-enhancing forces and negative destructive forces. |  |  |  |  |  |  |  |
| The body is essentially self-healing and the task of a health care provider is to assist in the healing process |  |  |  |  |  |  |  |
| A patient's symptoms should be regarded as a manifestation of general imbalance of dysfunction affecting the whole body |  |  |  |  |  |  |  |
| A patient's expectations, health beliefs and values should be integrated into the patient care process |  |  |  |  |  |  |  |
| Complementary therapies are a threat to public health |  |  |  |  |  |  |  |
| Treatments not tested in a scientifically recognized manner should be discouraged |  |  |  |  |  |  |  |
| Effects of complementary therapies are usually the result of a placebo effect |  |  |  |  |  |  |  |
| Complementary therapies include ideas and methods from which conventional medicine could benefit |  |  |  |  |  |  |  |
| Most complementary therapies stimulate the body's natural therapeutic powers |  |  |  |  |  |  |  |

1. **Use related CAM Beliefs**

(Select your option by tick mark)

| **Questions** | **Yes** | **No** |
| --- | --- | --- |
| Has somebody close to you had effective treatment from a complementary practitioner? |  |  |
| Is it only naive/gullible people who go to complementary practitioners? |  |  |
| Do you believe there are many “quacks” in complementary medicine? |  |  |
| Do you believe the most important factor of any treatment is its efficacy for cure? |  |  |
| Do you believe complementary medicine is more holistic than orthodox medicine? |  |  |
| Does someone close to you use a complementary practitioner? |  |  |
| Do you believe that complementary medicine is better than medicine to treat psychological illness? |  |  |

**SECTION: 5 ASSESSMENT OF ATTITUDES REGARDING CAM** (5- point Likert scale)

| **Statement** | **Strongly Disagree**  **1** | **Disagree**  **2** | **Neutral**  **3** | **Agree**  **4** | **Strongly Agree**  **5** |
| --- | --- | --- | --- | --- | --- |
| All practitioners of CAM should be medically qualified |  |  |  |  |  |
| I am interested in exploring new CAM modalities |  |  |  |  |  |
| Women have more tendency to CAM than men |  |  |  |  |  |
| On an average, practitioners of CAM make less money than other doctors |  |  |  |  |  |
| I believe in alternative approaches in health area |  |  |  |  |  |
| Most Practitioners of CAM receive a thorough training |  |  |  |  |  |
| Treating a condition using CAM is safer than using modern methods |  |  |  |  |  |
| You need to be “gifted” to carry out CAM |  |  |  |  |  |
| CAM has low status within medicine |  |  |  |  |  |
| CAM is only effective in treating minor complaints. |  |  |  |  |  |
| CAM is fairly unscientific. |  |  |  |  |  |
| CAM has advanced considerably in recent years in understanding of illness and diseases |  |  |  |  |  |
| Practitioners of CAM are more prepared to listen to their patients |  |  |  |  |  |
| Patients on CAM hardly ever get better |  |  |  |  |  |
| Despite considerable research, there are few applicable results in CAM. |  |  |  |  |  |
| CAM should be taught in medical school. |  |  |  |  |  |
| A surprising number of patients claim its effective at curing their illness |  |  |  |  |  |
| CAM is more cost-effective than modern medicine |  |  |  |  |  |
| The reason for the success of CAM is mainly due to treating the whole person |  |  |  |  |  |
| A doctor should know CAM methods |  |  |  |  |  |
| I believe that CAM may have positive effect on general health outcomes |  |  |  |  |  |
| It is important to have a basic understanding of CAM before using them |  |  |  |  |  |
| Patients may disregard or even avoid doctors and health care professionals who do not understand their health beliefs. |  |  |  |  |  |
| Herbal medicine is unsafe and ineffective |  |  |  |  |  |
| Self-care and interest in our own health is one reason that people are drawn to CAM |  |  |  |  |  |
| Providing information about herbal medicine is part of a pharmacist’s professional responsibility. |  |  |  |  |  |
| Providing information about herbal medicine is part of a doctor’s professional responsibility. |  |  |  |  |  |
| It is important to consult a health professional before using CAM. |  |  |  |  |  |

**SECTION: 6 SELF PERCEIVED EFFECTIVENESS OF CAM** (Select your option by tick mark)

| **Cam modality** | **Harmful** | **Useful** | **No opinion** |
| --- | --- | --- | --- |
| **Acupuncture** *Inserting fine needles through the skin* |  |  |  |
| **Aromatherapy** *Use of essential oils of plants* |  |  |  |
| **Ayurveda**  *Hikmat* |  |  |  |
| **Cupping/Hijama**  *Placing cups on skin to draw blood* |  |  |  |
| **Chiropractic** *Treating by pushing and moving bones in the spine and joints* |  |  |  |
| **Herbs**  *Herbal medicine* |  |  |  |
| **Homeopathy** *Herbal medicine* |  |  |  |
| **Hypnosis** *An artificially induced trance state resembling sleep* |  |  |  |
| **Meditation/Muraqba** *Act of spending time in quiet thought* |  |  |  |
| **Massage**  *Maalish* |  |  |  |
| **Oriental medicine** *Aims to restore balance of life energy Qi or Chee* |  |  |  |
| **Spiritual healing** *Prayer* |  |  |  |
| **Yoga**  *Practices to experience inner peace* |  |  |  |
